# Exploring the therapeutic potential of diterpenes in gastric cancer: Mechanisms, efficacy, and clinical prospects

**DOI:** 10.17305/bb.2024.10887

**Published:** 2024-08-15

**Authors:** Chenhui Ma, Lei Gao, Kewei Song, Baohong Gu, Bofang Wang, Weigao Pu, Hao Chen

**Affiliations:** 1 The Second Clinical Medical College, Lanzhou University, Lanzhou, China; 2 Gansu Provincial Key Laboratory of Environmental Oncology, Lanzhou, China; 3 Department of Tumor Surgery, Lanzhou University Second Hospital, Lanzhou, China

**Keywords:** Natural products, diterpenes, gastric cancer (GC), mechanisms, clinical prospects

## Abstract

Gastric cancer (GC) remains a significant global health challenge, particularly prevalent in East Asia. Despite advancements in various treatment modalities, the prognosis for patients, especially those in advanced stages, remains poor, highlighting the need for innovative therapeutic approaches. This review explores the promising potential of diterpenes, naturally occurring compounds with robust anticancer properties, derived from diverse sources, such as plants, marine organisms, and fungi. Diterpenes have shown the ability to influence reactive oxygen species (ROS) generation, ferroptosis, and autophagy, positioning them as attractive candidates for novel cancer therapies. This review delves into the mechanisms of action of diterpenes and their clinical implications for the treatment of GC. Additionally, it addresses the challenges in translating these compounds from preclinical studies to clinical applications, emphasizing the need for further research to enhance their therapeutic profiles and minimize potential side effects. The discussion underscores the importance of diterpenes in future anticancer strategies, particularly in the fight against GC.

## Introduction

As one of the most common malignancies worldwide, gastric cancer (GC) ranks as the fifth most prevalent cancer and the third leading cause of cancer-related deaths globally [1, 2]. The development of GC is often linked to various factors, including dietary habits, Helicobacter pylori infection, smoking, and genetic predispositions [3, 4]. Current treatment options, such as surgical resection [5], chemotherapy [6], targeted therapy, and immunotherapy [7], often yield limited success. This is primarily because most patients are diagnosed at an advanced stage, and these treatments frequently come with significant side effects. Thus, developing new therapeutic strategies and identifying novel treatment targets are critical for improving survival rates and the overall well-being of GC patients [8].

In the quest for new treatment strategies for GC, natural products have gained attention due to their unique biological activities [9]. Among them, diterpenoids have attracted widespread interest for their anticancer potential [10, 11]. Diterpenoids are derived from diverse sources, including plants, marine organisms, and insects, and possess unique chemical structures and biological activities [12–14]. They primarily inhibit tumor growth and metastasis by inducing apoptosis—programmed cell death in cancer cells [15, 16]. Additionally, they impede cell proliferation by disrupting the cell cycle and preventing cancer cell division [17, 18]. Moreover, diterpenoids can block key signaling pathways critical to cancer cell survival and metastasis [19], and disrupting these pathways effectively halts GC progression. Another key aspect of their anticancer activity is their ability to modulate the tumor microenvironment. Diterpenoids can modify the tumor surroundings, making them less conducive to cancer growth and more vulnerable to immune system attacks [20].

Preclinical studies, including both in vitro experiments and in vivo animal models, have been extensively used to assess the anticancer effects of diterpenoids [21]. These studies have provided substantial evidence supporting the efficacy of diterpenoids in inhibiting cancer cell growth and inducing apoptosis. In vitro experiments have shown the ability of these compounds to directly target and kill cancer cells, while in vivo studies have demonstrated their potential to reduce tumor size and inhibit metastasis in animal models. Additionally, pharmacokinetic and metabolic studies have been conducted to understand the absorption, distribution, metabolism, and excretion (ADME) of diterpenoids, which is critical for their potential clinical application [22].

The aim of this review is to provide a comprehensive discussion of the therapeutic potential of diterpenes in GC, exploring both their biochemical mechanisms and their implications for future clinical applications. The following sections will delve into the specific types of diterpenes, their mechanisms of action, and the current state of research in this promising field of oncology.

### Overview of GC

#### Global epidemiology and classification of GC

GC remains the third leading cause of cancer-related deaths worldwide, with the highest incidence rates observed in East Asia, particularly in China [2, 3, 23]. This regional disparity is often attributed to dietary habits, environmental factors, and the prevalence of *Helicobacter pylori* infection [4]. Despite advances in diagnosis and treatment, the absolute number of cases continues to be significant, largely due to the aging population and persistent cases in regions with underdeveloped healthcare infrastructures [4, 24, 25].

The classification of GC is critical for guiding both diagnosis and treatment and for understanding patient prognosis. GCs are primarily categorized into adenocarcinomatous and nonadenocarcinomatous types. The most prevalent type, adenocarcinoma [26], is further subdivided based on the Borrmann classification into four types, with type IV being characterized by the worst prognosis due to its diffuse infiltrative growth patterns. The Lauren classification system further differentiates adenocarcinomas into intestinal, diffuse, and mixed types—each with distinct pathological features linked to specific risk factors and disease progression patterns [27]. The intestinal type is often associated with chronic inflammation resulting from dietary factors and *H. pylori* infection, whereas the diffuse type is more influenced by genetic factors [28].

The Lauren classification [29] categorizes adenocarcinomas into intestinal, diffuse, and mixed types, thereby elucidating their histological characteristics and associated risk factors [30]. Intestinal adenocarcinomas are well-differentiated, form gland-like structures, and are frequently associated with diet and *H. pylori* infection. This type of adenocarcinoma is associated with extensive intestinal metaplasia and atrophic gastritis, suggesting that the cancer develops as a result of chronic inflammation. In contrast, the diffuse type of adenocarcinoma is characterized by poorly differentiated cells that spread diffusely and do not form glandular structures. Genetic factors, rather than environmental influences, are often associated with the diffuse type of adenocarcinoma [31]. Moreover, the WHO classification refines this further by identifying specific carcinoma subtypes, such as papillary, tubular, mucinous, and signet-ring cell carcinomas, each with unique implications for treatment and prognosis [32]. Understanding these classifications aids in tailoring treatment approaches, which is crucial, given the variability in survival rates and treatment responses among the different types of GC [33, 34].

### Clinical treatment

**Figure 1.**
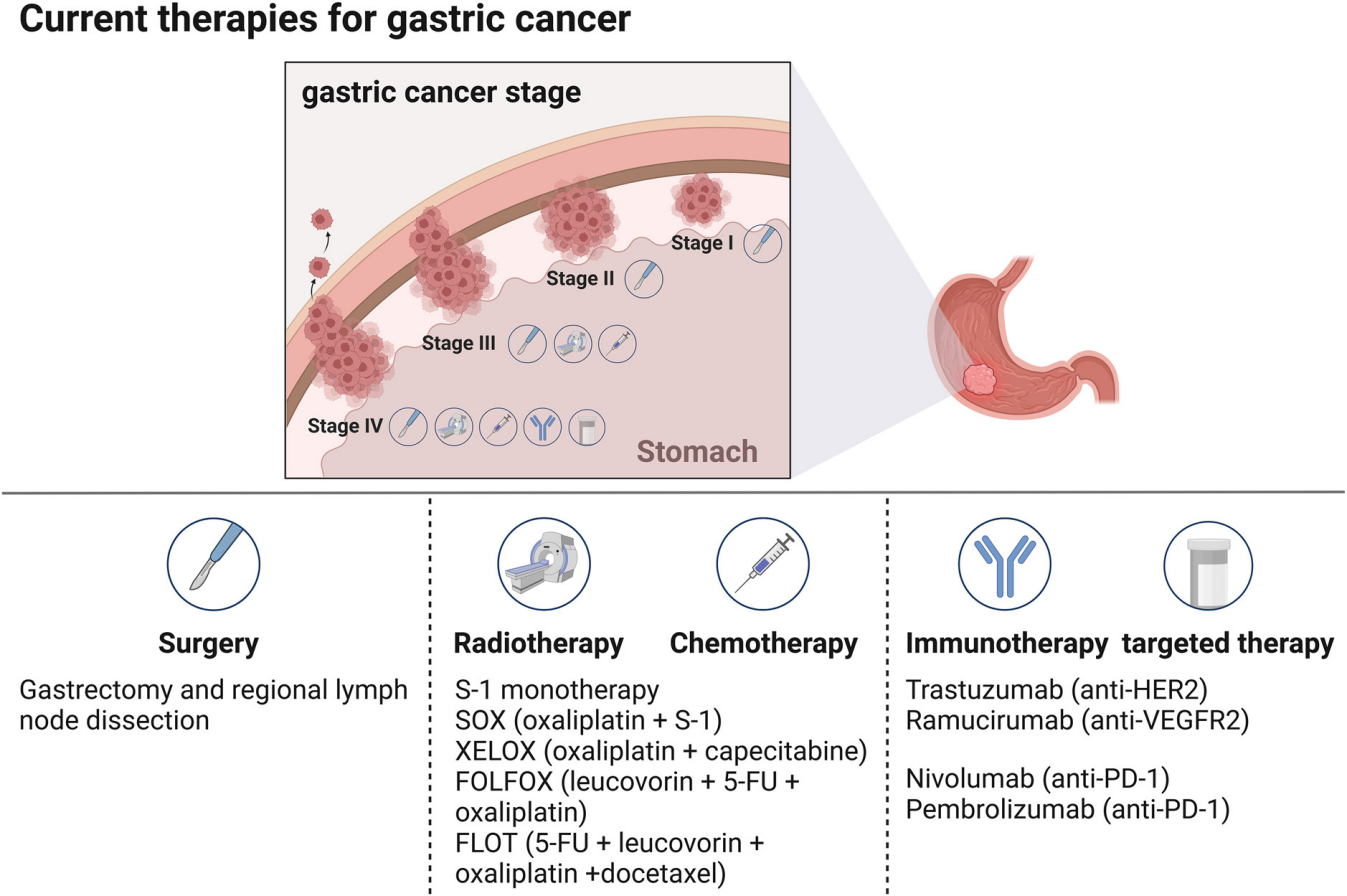
**Therapies for treating GC.** Current GC treatments are stage based, with therapeutic interventions varying according to the cancer stage. GC: Gastric cancer.

The treatment landscape for GC encompasses a range of therapeutic modalities, including surgery, radiation therapy, chemotherapy, and targeted therapy (Figure 1). Surgical intervention is the cornerstone of early-stage GC treatment and is often considered the most effective means of achieving a potential cure [8]. The extent of surgical intervention ranges from partial gastrectomy to total gastrectomy, depending on the location and size of the tumor, with radical gastrectomy and D2 lymphadenectomy being the standard procedures for resectable tumors [35, 36]. However, surgery alone is often insufficient for patients in advanced stages, necessitating additional treatments, such as chemotherapy and radiation therapy. Common regimens include platinum-based drugs and fluoropyrimidines, which are used to reduce tumor size before surgery and eliminate residual cancer cells afterward [37]. The development of targeted therapies, which specifically attack cancer cells by interacting with particular molecular markers, has been driven by advances in molecular biology. For example, trastuzumab targets HER2-positive GC and has shown improved outcomes when combined with chemotherapy [38]. Immunotherapy has also emerged as a promising treatment approach, enhancing the immune system’s ability to fight cancer. Agents, such as PD-1 and PD-L1 inhibitors have demonstrated potential benefits [33]. Despite the availability of these various treatment modalities, the overall prognosis for GC patients remains poor, especially for those diagnosed at advanced stages.

### Future directions of natural products in GC treatment

Despite the availability of diverse treatment modalities for GC, the prognosis remains poor, particularly for patients diagnosed at advanced stages. These findings underscore the urgent need for innovative therapeutic strategies to improve patient outcomes. Among the promising approaches, natural products derived from plants, marine organisms, and fungi offer significant potential due to their diverse bioactive properties.

Plant-derived natural compounds: Plant-based compounds are potent sources of anticancer agents. Polyphenols, including flavonoids, such as quercetin and catechins like epigallocatechin gallate (EGCG), and resveratrol, found in grapes and berries, are well-known for their anti-inflammatory and anticancer properties [39]. These compounds inhibit tumor growth and induce apoptosis by modulating critical signaling pathways, such as p53, NF-κB, and STAT3 [40]. Additionally, plant bioactive compounds have been reported to influence the dysregulation of miRNAs and the ubiquitin–proteasome pathway in cancer cells [41, 42]. Alkaloids such as berberine and piperine target multiple molecular mechanisms involved in cell cycle regulation and apoptosis, increasing the bioavailability of therapeutic compounds and displaying direct anticancer activity [43]. Terpenes, including monoterpenes and sesquiterpenes like artemisinin, along with triterpenes like ursolic acid, have been shown to inhibit tumor growth and promote apoptosis [44, 45]. Sulfur compounds, such as sulforaphane, influence gene expression and stimulate the production of detoxification enzymes. Glycosides, such as ginsenosides, not only modulate immune responses but also induce cancer cell death [46]. Moreover, these natural saponins are being explored as potential inhibitors of aquaporins, water channel proteins that play crucial roles in cellular processes related to cancer progression [47]. Marine-derived compounds: The ocean provides a vast repository of unique bioactive substances. Marine-derived polysaccharides, such as fucoidan, not only induce apoptosis and inhibit the proliferation of GC cells but also enhance the immune response, suggesting a multifaceted approach to cancer therapy [48]. Marine peptides have demonstrated potential in inducing apoptosis, inhibiting angiogenesis, and possessing antimetastatic properties [49]. Sesterterpenoids and diterpenoids from marine sources, such as sponges, stabilize microtubules, which are essential for controlling cell division. This prevents the proper segregation of chromosomes, ultimately leading to cancer cell death [50]. Marine alkaloids interact with DNA and inhibit topoisomerases, disrupting DNA synthesis and repair mechanisms [51]. Fungi-derived compounds: Fungi offer a wide range of effective compounds against GC [52]. Polysaccharides, such as lentinan from shiitake mushrooms and beta-glucans found in various fungal species enhance the immune system’s ability to fight cancer. PSK (Krestin), a protein-bound polysaccharide from turkey tail mushrooms, exhibits direct anticancer effects by inhibiting cell proliferation and inducing apoptosis. Lectins from common mushrooms such as Agaricus bisporus induce apoptosis by specifically binding to cancer cell membranes. Terpenoids such as ganoderic acids from reishi mushrooms inhibit tumor invasion and metastasis and are being explored to improve the efficacy of chemotherapy. Among these, diterpenes stand out for their potential to revolutionize GC treatment due to their ability to act synergistically with existing therapies. This synergy could reduce the required dosages of traditional chemotherapy, thereby minimizing toxicity. Furthermore, diterpenes have shown promise in overcoming drug resistance by targeting and modulating multiple pathways involved in cancer cell survival and resistance mechanisms. The exploration of diterpenes in GC treatment opens new avenues for research and holds the potential for significant breakthroughs in improving the efficacy of existing treatment protocols and developing novel therapeutic strategies. Their integration into GC treatment regimens offers a promising path forward, emphasizing the need to harness their full therapeutic potential to improve outcomes for patients battling this challenging disease.

### Diterpenes

Diterpenes are composed of four isoprene units with the molecular formula C20H32 and are synthesized by various organisms through the HMG-CoA reductase pathway. These compounds have gained prominence in medicinal chemistry and biology due to their diverse biological activities. They play a crucial role in the development of therapies for cancer, inflammation, and the prevention of osteoporosis. Many diterpenes and their derivatives are effective anticancer agents, capable of affecting multiple critical biological pathways. Taxanes, such as Taxol (paclitaxel) and its albumin-bound formulations, are known for their effectiveness in treating various cancers by stabilizing microtubule formation, thus inhibiting cell division [53, 54]. Triptolide, extracted from the Thunder God Vine, possesses potent immunosuppressive and anti-inflammatory properties and plays a significant role in cancer therapy by modulating multiple signaling pathways that support tumor growth and survival [55]. Oridonin, derived from the herb Rabdosia rubescens, induces apoptosis and inhibits cell proliferation, making it a promising candidate for cancer treatment [56]. Andrographolide, from Andrographis paniculata, is recognized for its anti-inflammatory and anticancer properties, potentially acting through mechanisms that alter the body’s immune response and directly curb cancer cell growth. Additionally, diterpenes from coffee, such as cafestol, kahweol, and caffeic acid, have been studied for their anticarcinogenic properties, particularly their ability to activate detoxifying enzymes and protect against oxidative stress [57].

#### Sources

Diterpenes are predominantly found in higher plants, where they serve as crucial chemotaxonomic markers [58]. For example, the Euphorbia species produces a diverse array of diterpenes, including jatrophane, ingenane, and pepluane, which have been extensively studied for their potent biological activities and potential therapeutic applications [58]. Fungi also contribute to the diversity of diterpenes, particularly through the production of indole diterpenes—compounds that combine a diterpenoid backbone with an indole structure—such as aflatrems and lolitrems, known for their neurotoxic and antimicrobial properties [59]. Moreover, marine ecosystems significantly expand the diversity of diterpenes, with marine sponges and other organisms synthesizing variants that perform protective functions similar to their terrestrial counterparts, such as defense against predators and microbial infection [60]. In addition to naturally occurring diterpene compounds, synthetic derivatives have been developed to optimize their pharmacological characteristics. These synthetic derivatives are engineered to enhance solubility, increase stability, and improve specificity toward biological targets [61]. For example, modifications to the diterpene structure can improve drug delivery mechanisms or reduce toxicity, making them more suitable for clinical application. Synthetic analogs of Taxol are a prime example, where alterations to the ester side chains or the core diterpene structure have enabled these agents to overcome drug resistance, thereby increasing their efficacy and minimizing adverse effects [62]. These advances underscore the crucial role of both natural and synthetic diterpenes in the development of new therapies, integrating natural product research with medicinal chemistry and biotechnology to address complex health challenges.

#### Diterpene classification

Diterpenes exhibit remarkable structural diversity, ranging from simple linear configurations to complex cyclic configurations (Table 1). This diversity significantly influences their biological functions and their applications in pharmacology and biotechnology. The simplest acyclic diterpenes, such as phytane and chromista, feature a straightforward carbon backbone without cyclic structures. Macrocyclic diterpenes, characterized by large ring structures formed by the joining of molecular ends, are more complex and are predominantly found in plant families such as Euphorbiaceae and Thymelaeaceae [58]. Bicyclic diterpenes, such as the labdane and abietane types, contain two fused rings, are prevalent in coniferous trees, and exhibit anti-inflammatory and antimicrobial properties. Tricyclic diterpenes, which feature three fused rings and include the cyathane and fusicoccane types, are derived from both fungal and marine sources and are known for their diverse biological activities [59, 60]. Tetracyclic diterpenes, with four fused rings, include crucial plant growth hormones such as gibberellins, along with structurally unique molecules, such as stemarene and guanacastane, which are renowned for their wide-ranging biological functions. Indole diterpenes, which possess a diterpenoid backbone with an indole structure, exhibit potent biological activities and are predominantly produced by filamentous fungi. This category includes variants such as the paxilline and nonpaxilline types. The structural diversity of diterpenes is further enhanced by modifications such as oxygen-containing functional groups (e.g., hydroxyls and ketones), esterifications, and other substituents, which significantly alter their chemical properties and biological effects. The intricate “6/6/7” ring systems of isopimarane and syn-pimarane diterpenes exemplify sophisticated biosynthetic pathways that contribute to the structural diversity of diterpenes. These compounds serve not only as fundamental bioactive agents but also as chemotaxonomic markers, highlighting their evolutionary significance and ecological roles [63]. The structural diversity of diterpenes is further augmented by various modifications: the incorporation of oxygen-containing functional groups, including hydroxyls and ketones; esterifications; and the addition of other substituents, which significantly impact their chemical properties and biological activities. Cystathionine-type diterpenes, with their tricyclic structures, exhibit various biological activities, including anti-inflammatory, cytotoxic, antibacterial, and antiviral properties. The complexity of their structure provides a versatile framework for interactions with various biological targets. Fusicoccane-type diterpenes, characterized by their tetracyclic skeletal structures, are typically found in fungal genera, such as Fusicoccum and Alternaria. The diverse biological activities of these compounds make them compelling subjects for pharmacological research. Harziene-type diterpenes, primarily identified in Trichoderma species, also possess tetracyclic structures and are notable for their unique carbon skeletons and wide range of biological activities, highlighting the potential of diterpenes as promising therapeutic agents [64].

**Table 1:** Classification and sources of diterpenoids

**Classifications**		**Sources**		**Example**
Acyclic diterpenes	Plantae	*Aphanamixis*	Aphanamixins A-F [9, 113], Nemoralisin [114]	
	Chromista	*Bifurcaria*	Eleganediol, Bifurcane [115]	
	Photosynthetic organisms	Phytol [116]		
Monocyclic diterpenes	Animalia	*Bovidae, Phasianidae*	Retinol [117]	
		*Alcyoniidae*	Cembrene A [118]	
	Plantae	*Asteraceae*	Tagetones A-B [119]	
Bicyclic diterpenes	Labdanes	Plantae	*Acanthaceae*	Andrographolide [120]
			*Lamiaceae*	Forskolin [121], Sclareol [122]
	Halimanes	Plantae	*Asteraceae, Lamiaceae, and Euphorbiaceae* [123]	/
	Clerodanes	Plantae	*Euphorbiaceae*	Calyculins [124]
	Casbane	Animalia	*Alcyoniidae*	Sinularcasbane [125]
		Plantae	*Euphorbiaceae*	Crotonitenone [126], Jolkinolide E [17]
Tricyclic diterpenoids	Abietanes	Plantae	*Pinaceae*	Abietic acid [65], Ferruginol [127]
			*Lamiaceae*	Carnosic acid [86]
	Pimaranes	Plantae	*Pinaceae*	Pimaric acid, Isopimaric acid [128]
	Cassanes	Plantae	*Fabaceae*	Caesalpin A-B [22]
Tetracyclic diterpenoids	Kaurane	Plantae	*Lamiaceae*	Oridonin [22]
			*Asteraceae*	Steviol [121]
	Trachylobane	Plantae	*Asteraceae*	6,19-Dihydroxy-ent-trachiloban-17-oic acid [121]
	Aphidicolane	Fungi	*Nectriaceae*	Aphidicolin [60]
	Stemodane	Plantae	*Scrophulariaceae*	Stemodin [129]
	Stemarane	Plantae	*Scrophulariaceae*	Stemarin [128]
	Beyerane	Plantae	*Lamiaceae*	Beyeric acid [130]
	Atisane	Plantae	*Ranunculaceae*	Atisine [130]
	Scopadulane	Plantae	*Scrophulariaceae*	Scopadulcic acid A [130]
	Gibberellane	Fungi	*Nectriaceae*	Gibberellic acid [130]
	Jatrophane	Plantae	*Euphorbiaceae*	Jatrophone [131]
	Ingenane	Plantae	*Euphorbiaceae*	Ingenol [131]
	Tigliane	Plantae	*Euphorbiaceae*	Phorbol [131]
	Taxane	Plantae	*Taxaceae*	Taxane [54]
	Daphnane	Plantae	*Thymelaeaceae*	Daphnane [132]
	Ppolycyclic—cembrane	Animalia	*Alcyoniidae*	Cembranoids [118]
Others			/	

Various diterpenoids are categorized according to their chemical structure, ranging from acyclic to tetracyclic, with their respective natural sources highlighted. This review includes specific examples of each diterpenoid to provide a clear reference for their potential applications and biological significance. Each classification is further detailed by the type of organisms (plants, animals, or fungi) that produce these compounds, underscoring the diversity of diterpenoids in nature. This comprehensive overview aids in understanding the wide distribution and ecological roles of these chemically and functionally diverse molecules.

### Mechanism of action of diterpenes against GC

**Figure 2.**
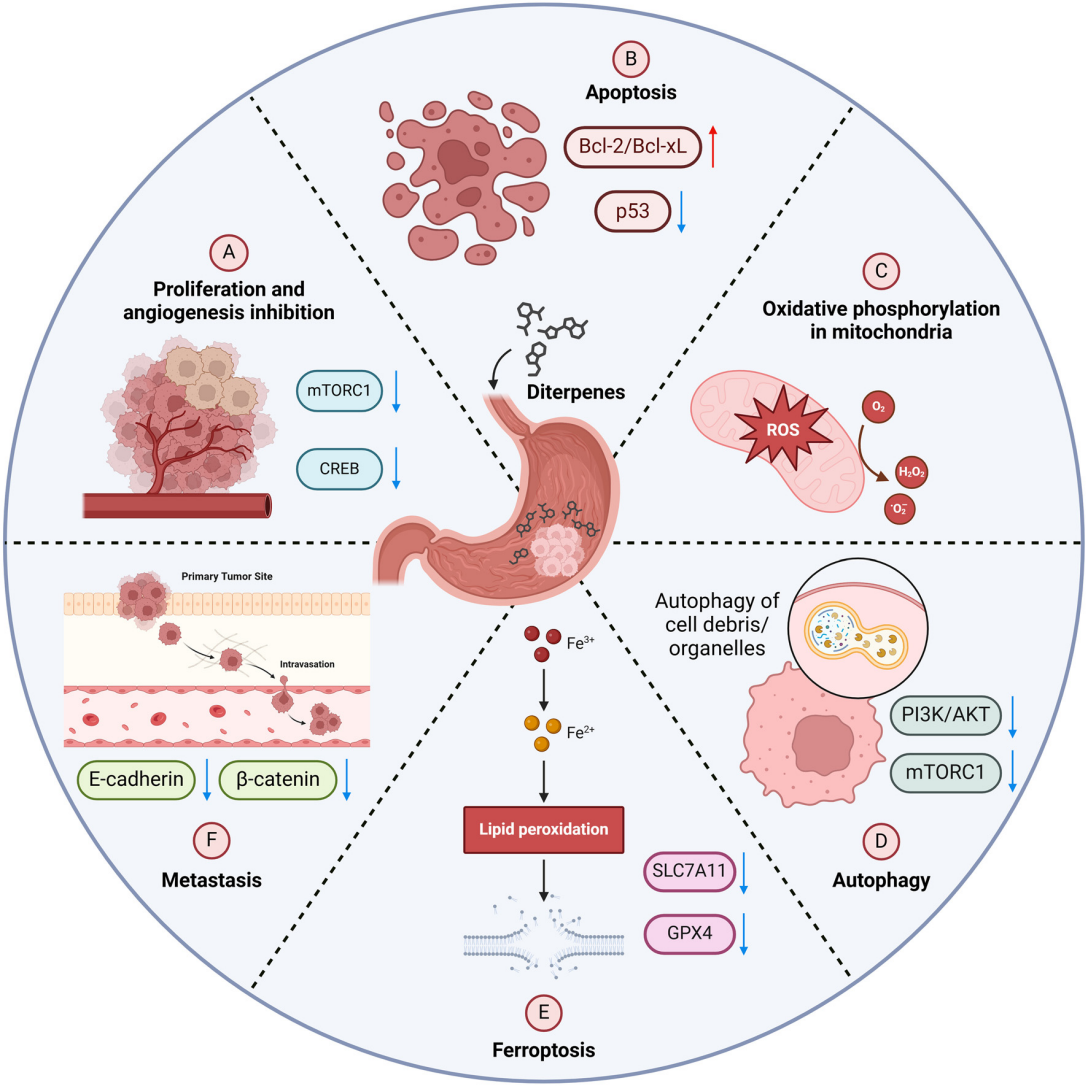
**Diterpenes in GC therapy.** This figure illustrates how diterpenes target GC through multiple pathways. Diterpenes promote apoptosis by activating p53 and inhibiting Bcl-2/Bcl-xl, reduce cell proliferation via mTORC1 and CREB, and induce autophagy and ferroptosis by disrupting PI3K/AKT and increasing lipid peroxidation, respectively. Increased ROS production further aids in cancer cell death. This figure highlights the comprehensive anticancer potential of diterpenes. GC: Gastric cancer.

Diterpenes target various critical aspects of GC cell biology and tumor progression. Known for their diverse biological effects (Figure 2) [65], their multifunctional nature allows them to interact with and modify key molecular mechanisms, making them valuable agents in the fight against GC (Table 2).

#### Induction of oxidative stress

Elevated reactive oxygen species (ROS) play a dual role in cellular biology. While ROS are critical for defending against pathogens, an imbalance can induce oxidative damage, leading to cancer cell apoptosis. This oxidative stress response is a key therapeutic target of diterpenes in cancer treatment. Diterpenes induce oxidative stress by damaging DNA, lipids, and proteins, triggering a cascade of pro-apoptotic signals while simultaneously inhibiting cell survival pathways.

**Figure 3.**
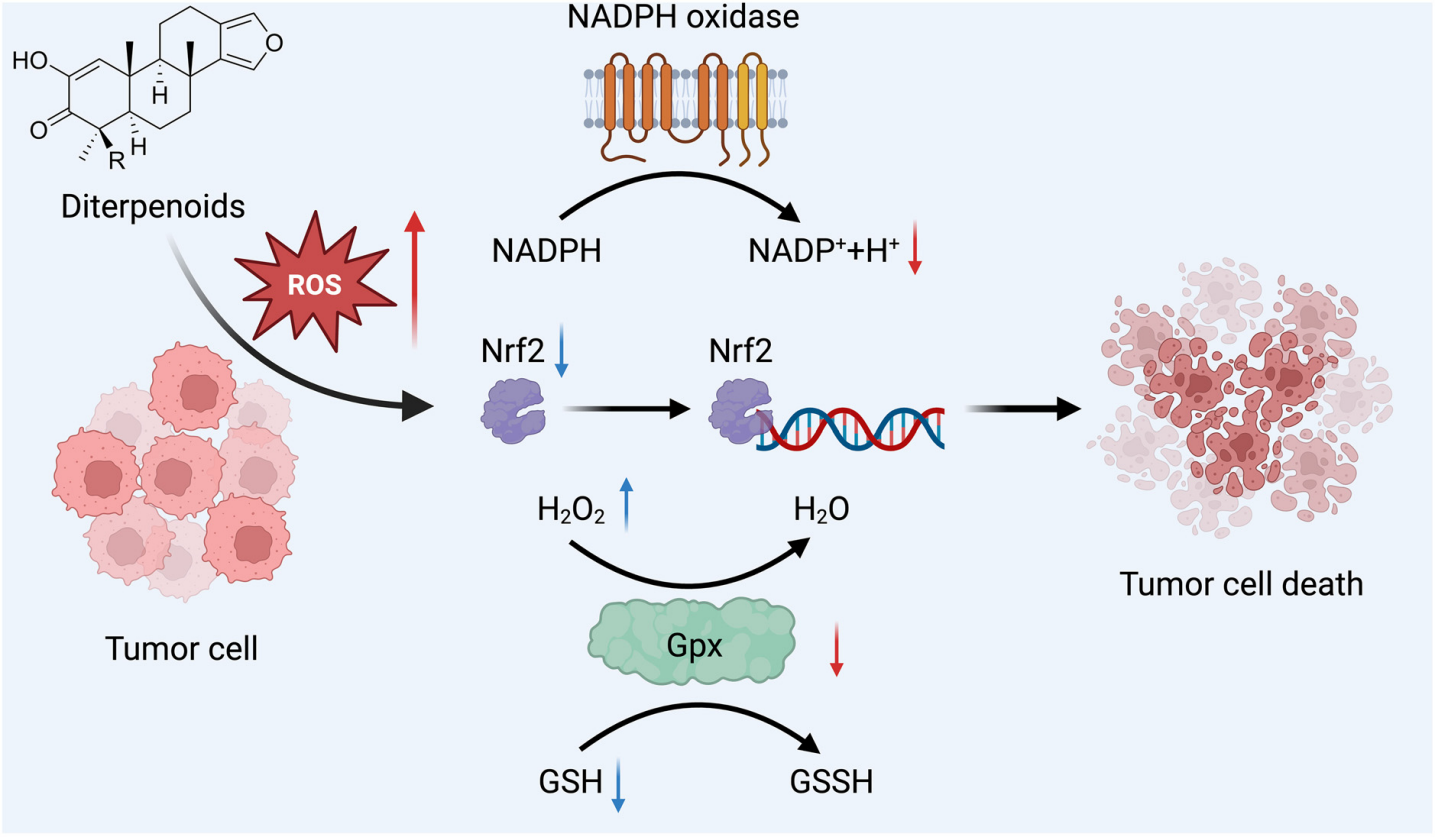
**Diterpenes in GC therapy.** Mechanism of ROS elevation by diterpenes. Diterpenes increase ROS, activating the NRF2 and HIF1α pathways and impacting glutathione dynamics, leading to redox balance disruption and subsequent GC cell death. GC: Gastric cancer; ROS: Reactive oxygen species.

Diterpenes influence cancer cell biology primarily through two mechanisms: direct induction of ROS and impairment of mitochondrial function. Elevated ROS disrupts mitochondrial integrity, releasing proapoptotic factors, activating caspases, and leading to diterpene-induced cell death. Some diterpenes directly increase ROS levels within cancer cells (Figure 3). For example, sugiol has been demonstrated to significantly increase intracellular ROS levels in SNU-5 GC cells, leading to cell death [66]. Compounds, such as B19, increase ROS levels, triggering ER stress and mitochondrial dysfunction, leading to GC cell apoptosis [67].

Diterpenes can also compromise cellular antioxidant systems. For instance, auranofin (AF) induces ROS-mediated apoptosis by inhibiting thioredoxin reductase 1 (TrxR1) activity, thereby increasing oxidative stress in GC cells. This inhibition disrupts the redox balance, enhancing the sensitivity of cancer cells to oxidative damage [68]. Furthermore, combining diterpenes with other agents can amplify ROS production and increase cytotoxicity. For example, the proliferation and migration of renal cancer cells are synergistically inhibited by kahweol acetate and cafestol, both derived from coffee, through increased ROS levels. This approach could similarly be utilized in GC therapy to enhance the efficacy of diterpenes [69].

**Table 2:** Multifaceted effects of diterpenes on GC

**Compound name**	**Cell line**	**Mechanism**	**Signaling pathway**
Tanshinones	SGC-7901 HUVECs	Angiogenesis *↓*	Suppresses the PI3K/Akt/mTOR signaling pathway VEGF *↓* [133]
Tanshinone IIA	AGS cells	Apoptosis and cell cycle arrest	CDC2 and cyclin B1 expression *↓* TNF-α, FAS, caspase-8, and caspase-3 *↑* [134]
Dehydroabietic acid	AGS MKN-28 SNU-216 SNU-601 SNU-668 YCC-2	Apoptosis	Inhibits survivin [73]
Sinulariolide	AGS NCI-N87	Migration and invasion *↓*	FAK/PI3K/AKT/mT OR MAPKs [135]
Oridonin	MKN-28	Migration and invasion *↓*	Inhibits ezrin [136]
Sugiol	SNU-5 SNU-1	ROS *↑* Cell cycle arrest	Inhibits STAT3 signaling [66]
Curcumin derivative B19	SGC-7901 BGC-823 KATO III	ROS *↑*	Inhibits TrxR1 enzyme activity [67]
Auranofin	SGC-7901 BGC-823 KATO III	ROS *↑*	Inhibits TrxR1 activity [68]
Carnosol	BGC803 and SGC-7901	Apoptosis and cell cycle arrest	Inhibits the RSKs-CREB signaling pathway [86]
Jolkinolide B	MKN45 cell	Apoptosis and cell cycle arrest	Activates the ATR-CHK1-CDC25A -Cdk2 signaling pathway [74]

Enumerating their effects across distinct cell lines, the key biological processes they target, and the signaling pathways they influence, this information emphasizes the potential utility of diterpenes in precision GC therapeutics. GC: Gastric cancer.

### Induction of cell cycle arrest and apoptosis

Apoptosis, a programmed cell death mechanism, plays a vital role in the regulation and turnover of biological tissues. This process is driven by a balance between oncogene activation and tumor suppressor gene deactivation, leading to abnormal cell proliferation and differentiation. The apoptotic process is pivotal for preventing tumorigenesis and can be categorized into two main pathways: the extrinsic pathway and the intrinsic pathway [70]. The extrinsic pathway is initiated by transmembrane receptors, such as TNF receptors, which possess cysteine-rich extracellular domains and a cytoplasmic “death domain” essential for transmitting apoptotic signals. The binding of death ligands, including FasL/FasR and Apo2L/TRAIL receptors (DR4 and DR5), to their respective receptors facilitates the recruitment of adaptor proteins, leading to the formation of a death-inducing signaling complex (DISC). This complex activates Caspase-8/3, ultimately resulting in apoptosis. Mutations in the death domain can disrupt this pathway, causing receptor dysfunction.

The intrinsic pathway, which is triggered by internal cellular stress, increases mitochondrial permeability, releasing proapoptotic proteins such as cytochrome c, AIF, and other regulatory proteins into the cytosol. Cytochrome c interacts with Apaf-1 to form an apoptosome, activating Caspase-9, which subsequently activates Caspase-3. This cascade leads to the cleavage of PARP, a critical DNA repair enzyme, resulting in DNA fragmentation and cell death.

Diterpenes, a class of naturally occurring compounds, have demonstrated significant anticancer effects through diverse mechanisms of action. Karmakar et al. discovered that a pimarane diterpene from *Boesenbergia pandurata* induces apoptosis in TRAIL-resistant AGS and noncancerous HEK293 cells by modulating the expression of death receptors (DR4 and DR5), proapoptotic proteins (p53, Fas, CHOP, Bak), and caspases while concurrently downregulating antiapoptotic proteins such as Bcl-2 and c-FLIP [71]. Scopadulciol, derived from Scoparia dulcis, targets AGS human gastric adenocarcinoma cells, inducing apoptosis via the TRAIL pathway with selectivity and efficacy [72].

Diterpenes also affect cell cycle regulation by increasing the expression of cyclin-dependent kinase inhibitors such as p21 and p27, which are crucial for controlling cell cycle progression. These inhibitors bind to and inhibit cyclin-CDK complexes, thereby blocking the transition between cell cycle phases. For example, dehydroabietic acid (DAA) induces cell cycle arrest and apoptosis in GC cells by downregulating survivin, a protein that inhibits apoptosis, and increasing the levels of cleaved caspase-3, essential for apoptosis activation [73]. Jolkinolide B (JB), an ent-abietane-type diterpenoid from Euphorbia fischeriana, causes DNA damage in GC MKN45 cells. JB induces S-phase cell cycle arrest by activating the ATR-CHK1-CDC25A-Cdk2 signaling pathway, inhibiting cell cycle progression and promoting apoptosis via the mitochondrial pathway [74]. Another diterpene, sageone, from Rosmarinus officinalis, induces apoptosis in SNU-1 human GC cells and enhances cisplatin’s cytotoxic effects. This is achieved through increased levels of cleaved caspase-3/9 and ADP-ribose PARP, which are essential in apoptosis execution [75].

#### Inhibition of cell proliferation and angiogenesis

Diterpenes inhibit GC cell proliferation by targeting key signaling pathways controlling cell growth, such as the MAPK/ERK and PI3K/Akt pathways. By disrupting these pathways, diterpenes reduce the proliferative capacity of cancer cells, impeding tumor growth. Tanshinones, diterpenoids derived from Salvia miltiorrhiza, inhibit GC angiogenesis and cell proliferation through the PI3K/Akt/mTOR signaling pathway. Cucurbitacins, a class of triterpenoids, inhibit the Ras/Raf/ERK/MMP9 signaling pathway to combat GC. Oridonin, a diterpenoid isolated from Rabdosia rubescens, inhibits GC cell proliferation by targeting the TNF-α/androgen receptor/TGF-β signaling pathway, altering cell morphology and causing nuclear fragmentation, leading to reduced cell viability and proliferation [76].

#### Inhibition of cell migration, invasion, and metastasis

Cell migration is an essential process for the development and maintenance of multicellular organisms, and errors in this process can lead to tumor formation and metastasis. External chemical or mechanical signals can trigger cell migration [77], providing opportunities for strategic cancer treatment interventions. Tanshinone IIA has been shown to effectively inhibit GC cell migration by downregulating key proteins involved in the migration process, such as NF-κB-p65, COX-2, and MMP-2, -7, and -9. These proteins play significant roles in cell adhesion, extracellular matrix degradation, and facilitating cellular movements essential for invasion and metastasis. By targeting these molecules, tanshinone IIA acts as a potent inhibitor of cancer cell invasion and metastasis, offering a promising approach for limiting cancer progression [78]. Triptolide inhibits the EMT phenotype, linked to increased migration, invasion, and metastasis, in Taxol-resistant lung cancer cells [79]. Oridonin’s antimetastatic effects include the inhibition of key signaling pathways, such as the mTOR, HIF-1α/VEGF, and Notch pathways, along with the downregulation of proteins involved in EMT, invasion, and angiogenesis in various cancer types [80]. A study on the GC cell line HGC-27 revealed that oridonin treatment inhibited colony formation, linked to metastatic potential [81]. Oridonin also inhibits tumor angiogenesis, associated with metastasis [82]. Andrographolide treatment on the GC cell line SGC-7901 decreased cell survival, migration, and invasion in a dose-dependent manner by inhibiting MMP-2 and MMP-9 activity and upregulating tissue inhibitors of MMPs (TIMP-1 and TIMP-2) [83]. Transwell assays demonstrated that PTX treatment inhibited the migration and invasion of human GC cell lines SGC-7901 and MKN-45 [84, 85]. Carnosol suppressed the anchorage-independent growth of GC cell lines SGC-7901 and BGC803, and this is linked to metastatic potential. Carnosol also inhibited gastric tumor growth in patient-derived xenografts in a mouse model [86].

#### Modulation of autophagy

Autophagy, a type of programmed cell death, is crucial in cancer research due to its role in degrading and recycling cellular components. This process is pivotal in cancer biology, influencing tumor progression and therapeutic response. As a fundamental biological mechanism in both growth and development, autophagy also facilitates tumor cell death. Normal autophagic activity is essential for maintaining cellular homeostasis, and its dysregulation can contribute to tumorigenesis [87-89].

Diterpenes modulate autophagy by targeting various molecular pathways, particularly the PI3K/Akt/mTOR pathway, which serves as a central regulator of this process. Diterpenes inhibit this pathway to initiate autophagy in cancer cells, increasing the turnover of damaged organelles and proteins. Depending on the context and degree of autophagy activation, this can lead to either the survival or death of cancer cells. Jaridon 6, a novel diterpene extracted from *Rabdosia rubescens* (Hemsl.) Hara, has the potential to combat drug resistance in GC. Jaridon 6 inhibits the proliferation of drug-resistant GC cells by suppressing SIRT1 and inducing autophagy via a mechanism involving inhibition of the PI3K–AKT pathway [84]. Similarly, GC cell chemosensitivity is enhanced by tanshinone diterpenes, such as tanshinone IIA, which is isolated from *Salvia miltiorrhiza*. This enhancement is achieved by inducing autophagy through the inhibition of the PI3K/Akt/mTOR signaling pathway. This modulation helps counteract chemotherapy resistance, demonstrating the potential of diterpenes to improve therapeutic outcomes in GC patients [85].

#### Induction of ferroptosis

Ferroptosis is a form of programmed cell death characterized by severe lipid peroxidation that leads to the destruction of cell membranes [90]. Unlike other modes of cell death, ferroptosis has unique biochemical and morphological characteristics [91]. Biochemically, ferroptosis involves significant iron accumulation, lipid peroxidation, and elevated levels of toxic lipid peroxidation products, such as malondialdehyde (MDA) and 4-hydroxynonenal (4-HNE) [92]. Morphologically, cells undergoing ferroptosis display significant alterations, such as swollen or reduced mitochondria, increased membrane density, and diminished or decreased cristae density [93]. Ferroptosis, a recently identified form of cell death, is characterized by intracellular iron overload and lipid peroxidation within the cell membrane. Growing evidence indicates that ferroptosis is intricately associated with various physiological and pathological processes, especially in cancer. For example, in GC, the expression levels of genes related to ferroptosis, such as ferroptosis suppressor protein 1 and CDGSH iron–sulfur domain 1, which are biomarkers of poor prognosis for patients with GC, are extremely high; these genes are promising therapeutic targets for future GC treatment [94].

Various natural products with biological activity can exert anticancer effects on cancer cells by initiating and executing the ferroptosis process. For example, the diterpenoid kayadiol, by activating p53, downregulates SLC7A11 and GPX4 expression, inducing ferroptosis and inhibiting the proliferation of natural killer T-cell lymphoma (NKTCL) cells [95]. In mammalian cells, the synthesis of glutathione (GSH) is facilitated by glutamate cysteine ligase (GCL) and glutathione synthetase (GSS). The formation of γ-glutamylcysteine from glutamate and cysteine is catalyzed by GCL, and GSS subsequently adds glycine to complete the synthesis of GSH [96]. In the roots of Actinidia valvata Dunn, corosolic acid promotes the ubiquitination of GSS by increasing the expression of homocysteine-inducible ER protein with ubiquitin-like domain 1 (HERPUD1). This process impairs GSH synthesis and induces ferroptosis in liver cancer cells [97]. Consistent with in vivo studies showing that corosolic acid inhibits tumor growth, this effect is achieved by promoting HERPUD1-mediated ferroptosis [97].

The SLC7A11/GSH/GPX4 system is highlighted as a critical target through which terpenoids inhibit cancer progression, underscoring the importance of these findings. Additionally, the ent-kaurane diterpenoid derivative Jiyuan oridonin A2 induces ferroptosis in GC cells by decreasing GPX4 levels and causing ferrous iron accumulation. This is another mechanism by which diterpenoids target redox balance to overcome cisplatin resistance [98].

### Challenges and prospects

#### Challenges

Despite the recognized potential of diterpenoids as anticancer agents, their application in the treatment of GC presents substantial challenges due to factors such as low stability, solubility, poor bioavailability, rapid metabolism, and significant toxicity. Preclinical studies have explored numerous molecular targets and therapeutic pathways of diterpenes for treating GC, laying a solid foundation for human trials. However, translating these findings into clinical settings is fraught with hurdles, primarily concerning safety and toxicity profiles. For example, despite its potent anticancer effects, triptolide is associated with severe adverse effects, including gastrointestinal disturbances, hematological toxicity, and potential nephrotoxicity [99].

To address these challenges, rigorous clinical trials are crucial. These trials assess not only the efficacy and safety of diterpenes but also their impact on patients’ quality of life. A key example is the clinical deployment of minnelide, a water-soluble prodrug of triptolide designed to mitigate some of the harsh effects of the parent compound while preserving its therapeutic efficacy. A phase I trial of minnelide, involving patients with advanced gastrointestinal cancers, including GC, was conducted as an open-label, single-center, dose-escalation study. This trial highlighted a manageable safety profile with a disease control rate of 54%. However, severe cerebellar toxicity in some patients highlights ongoing concerns about diterpene toxicity [100].

Despite these challenges, such trials are vital for optimizing dosing guidelines, managing side effects, and ultimately enhancing patient outcomes. Therefore, the clinical translation of diterpenoid compounds for GC treatment faces numerous challenges, including the need to optimize dosing, manage side effects, and improve patient outcomes. These challenges underscore that although diterpene-based therapies may help delay disease progression, significant work remains to effectively manage side effects and verify long-term survival benefits. Ensuring that these therapies can be safely integrated into existing cancer treatment regimens requires continuous research and meticulous adjustments during clinical trials and drug development.

**Figure 4.**
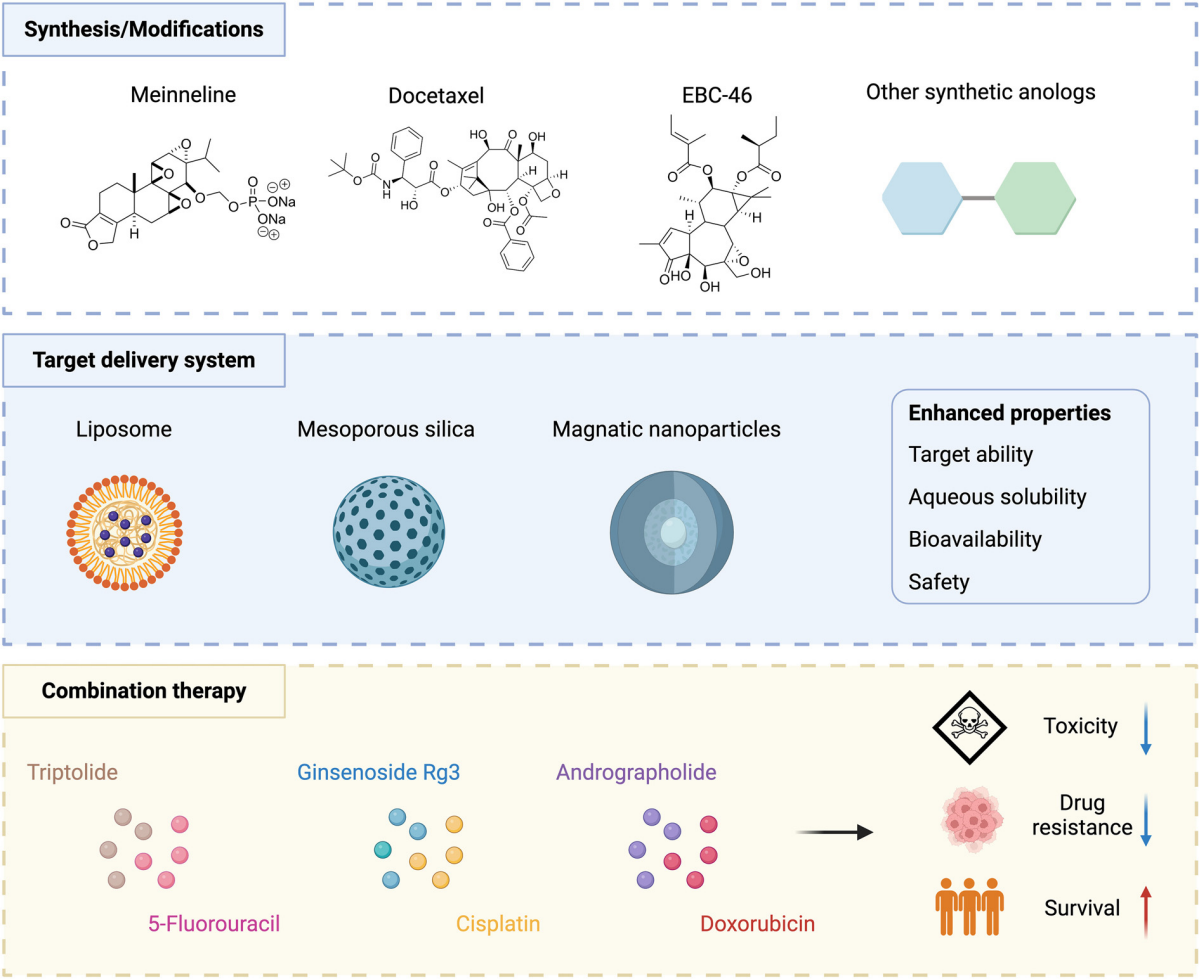
**Overview of current and emerging research on diterpenes.** Advancements in molecular modifications (e.g., minnelide, docetaxel, and EBC-46), integration with nanodelivery systems (e.g., liposomes, mesoporous silica, and magnetic nanoparticles), and combinations with traditional cancer therapies (e.g., triptolide with 5-fluorouracil, ginsenoside Rg3 with cisplatin, and andrographolide with doxorubicin) aim to enhance diterpene efficacy and delivery in GC treatment, focusing on improved outcomes and reduced toxicity. GC: Gastric cancer.

#### Prospects

As previously noted, the clinical application of diterpenoids in treating GC is limited by challenges, such as low stability, poor solubility, rapid metabolism, and significant toxicity. To address these challenges and enhance their therapeutic potential, substantial efforts are directed toward developing synthetic derivatives that retain the anticancer properties of natural diterpenoids while mitigating their drawbacks.

To overcome the toxicity issues associated with natural triptolide, minnelide, a water-soluble prodrug, has been developed. Clinical trials focus on establishing safe dosing guidelines and managing adverse effects to maximize therapeutic efficacy while minimizing side effects [101]. Similarly, docetaxel, a semisynthetic derivative of paclitaxel, which is naturally derived from the bark of the Pacific Yew tree, incorporates a hydroxyl functional group that enhances its solubility and therapeutic efficacy. Compared to its parent compound, docetaxel is widely used against various cancers and has superior pharmacokinetics [102]. Additionally, BC-46, originally derived from rare blushwood trees, faced significant challenges due to the rarity of the source. Advances in synthetic production techniques have made it possible to produce it on a scalable and sustainable basis, thus overcoming previous supply limitations [103]. Moreover, forskolin, a diterpene from Indian coleus, and its derivatives are known for their potent activation of adenylyl cyclase, which leads to increased levels of cAMP in various cell types. Enhancements, such as the addition of hydroxyl groups, have improved forskolin’s solubility and interaction with adenylyl cyclase, thereby increasing its biological efficacy [104]. To optimize the efficacy of diterpenes in cancer treatment and overcome challenges, such as selective toxicity, poor bioavailability, and rapid metabolism, advanced drug delivery systems are essential. Nanotechnologies, such as nanoparticles and liposomes, enhance the stability and targeted delivery of diterpenes, ensuring controlled release directly at tumor sites while minimizing exposure to healthy cells. This targeted approach not only maximizes therapeutic benefits but also extends the presence of diterpenes in the system. For example, encapsulating triptolide in liposomes enhances its bioavailability and reduces systemic toxicity [105]. These liposomes are engineered to release their payload specifically at the tumor site, optimizing drug efficacy and minimizing side effects. Similarly, poly(ethylene glycol)-block-poly(ɛ-caprolactone) nanoparticle micelles have been used to increase the bioavailability of triptolide, significantly enhancing its therapeutic potential while mitigating systemic toxicity [106]. Moreover, magnetic nanoparticles offer a targeted approach by using an external magnetic field to direct diterpenes precisely to tumor sites, which is especially beneficial for reaching tumors that are otherwise difficult to access with conventional methods [107]. Once localized, these nanoparticles provide controlled drug release, concentrating the treatment on the tumor and sparing healthy tissues. Additionally, mesoporous silica nanoparticles, known for their large surface areas and adjustable pore sizes, have high drug loading capacities. These nanoparticles can be further modified with specific ligands to target tumor markers, improving the delivery efficiency of diterpenes to cancer cells [108]. Integrating diterpenes with conventional anticancer therapies offers a promising strategy to increase the efficacy of cancer treatments and mitigate the development of resistance. This approach harnesses the ability of diterpenes to sensitize cancer cells to established chemotherapy drugs, such as platinum-based drugs and fluoropyrimidines, improving tumor reduction and extending progression-free survival (PFS). For example, preclinical studies have demonstrated that combining triptolide, from which the prodrug minnelide is derived, with cisplatin significantly increases apoptosis in GC cells, enhancing therapeutic outcomes through synergistic effects [109]. In clinical settings, the incorporation of diterpenes into treatment protocols primarily aims to overcome drug resistance, a significant challenge in advanced GC therapy. Trials combining diterpenes with other agents, such as docetaxel and ramucirumab, have shown promising results, improving patient responses and survival rates while maintaining manageable safety profiles [110, 111]. However, further comprehensive studies are necessary to fully understand the long-term benefits and potential toxicities of these combinations. Moreover, paclitaxel has been effectively utilized as a second-line therapy when combined with targeted treatments. A meta-analysis involving 1574 patients with advanced GC indicated that adding targeted therapies to paclitaxel improved not only PFS but also overall survival (OS), despite the increased occurrence of adverse events such as neutropenia and fatigue [112] (Figure 4). The future of diterpene research in cancer therapy centers on the development of new compounds, innovative drug delivery systems, and strategic combinations with existing anticancer agents, all aiming to optimize their therapeutic potential. Success in these areas requires precise and comprehensive clinical trials that focus on validating the efficacy and safety of diterpenes, necessitating the careful selection of patient populations, determination of optimal dosages, and rigorous assessment of long-term effects and side effects. Furthermore, a deeper understanding of the molecular mechanisms through which diterpenes act is crucial. Employing advanced technologies such as genomics, proteomics, and metabolomics will provide essential insights into their interactions with biomolecules, assisting in crafting more targeted and effective treatment protocols.

## Conclusion

Research on diterpenes has illuminated their remarkable potential in oncology, particularly for the treatment of GC. These natural compounds are known for their robust anticancer effects, such as inducing apoptosis, inhibiting tumor cell migration and invasion, and modulating the tumor microenvironment. Notably, their ability to prevent cancer metastasis represents a paradigm shift and presents a novel therapeutic opportunity for patients afflicted with GC. This review underscores the innovative aspects of diterpene research, particularly its role in regulating underexplored mechanisms, such as ferroptosis and autophagy, which could redefine therapeutic strategies for GC.

Despite the compelling potential of diterpenes, their clinical application faces significant hurdles, including low bioavailability, unresolved safety and efficacy concerns, and an incomplete understanding of their mechanisms of action. Addressing these challenges through focused and continuous research is crucial. A deeper understanding of the molecular interactions of diterpenes and advancements in their formulation and delivery are essential to harness their full therapeutic potential. By overcoming these barriers, diterpenes could become central to advanced GC treatment regimens and potentially improve the clinical outcomes of patients with this challenging disease, leading to significant advancements in medical oncology.
